# Supplementary material for: Optimization of Feed Components to Improve Hermetia illucens Growth and Development of Oil Extractor to Produce Biodiesel
Source: Animals (Basel). 2021 Sep 1;11(9):2573. doi: 10.3390/ani11092573 (PMC8470524; doi:10.3390/ani11092573)
Supplement: Supplementary file 1 [file animals-11-02573-s001.zip › animals-1290141-supplementary.pdf]

# Supplementary information

**Table S1.** The effect of press temperature on the oil extraction yield of the developed extractor.

| **Press Temperature (°C)** | **Yield (%)** |
|---|---|
| 150 | 35.3 ± 2.7 |
| 120 | 39.8 ± 1.3 |

HIL were dried by microwave at 18 kw for 6 min. Then, crude oil was extracted by the developed oil extractor at 120 °C or 150 °C. The crude oil was filtered to remove biomass and then the yield was determined.

**Table S2.** Nutritional components in crude oil extracted from HIL dried by microwave and drying oven.

| Nutrition (%) | Drying Method | |
|---|---|---|
| | Drying Oven | Microwave |
| Moisture | 0.59 | 0.38 |
| Carbohydrate | 4.06 | 1.98 |
| Crude protein | 8.78 | 8.24 |
| Crude fat | 85.20 | 88.21 |
| Crude ash | 1.37 | 1.19 |
| Crude fiber | 10.36 | 10.59 |

HIL were dried by drying oven at 70 °C and microwave at 18 kw for 6 h and 6 min, respectively. Then, crude oil was extracted by the developed oil extractor at 120℃.
